# Higher HbA1c‐Hemoglobin Ratio Was Associated With Poor Outcome in Endovascular Thrombectomy Patients

**DOI:** 10.1155/bn/6668235

**Published:** 2026-03-13

**Authors:** Feng Gao, Shuaiyu Chen, Xingjing Guo, Weiyao Xu, Chengfeng Guan, Shiyao Wang, Yanyan Shi, Shuo Wang

**Affiliations:** ^1^ Department of Radiology, Nanjing Jiangning Hospital, Nanjing Medical University, Nanjing, Jiangsu, China, njmu.edu.cn; ^2^ Department of Neurology, Nanjing First Hospital, Nanjing Medical University, Nanjing, Jiangsu, China, njmu.edu.cn; ^3^ Department of Physiology, Shandong Medical College, Jinan, Shandong, China, sdu.edu.cn; ^4^ Department of Cardiology, Nanjing First Hospital, Nanjing Medical University, Nanjing, Jiangsu, China, njmu.edu.cn

**Keywords:** cerebral infarction, elevated blood glucose, HHR, mechanical thrombectomy

## Abstract

**Background and Objectives:**

Recent studies have identified the HbA1c‐to‐hemoglobin ratio (HHR) as a potential indicator for increased mortality from all causes and cardiovascular diseases. This investigation sought to determine whether HHR could serve as a prognostic marker for neurological function at 90 days in patients undergoing endovascular thrombectomy procedures.

**Methodology:**

This study performed a retrospective evaluation of patients undergoing endovascular treatment (EVT) at Nanjing First Hospital from April 2022 to June 2024. The HHR was determined by dividing HbA1c levels by hemoglobin values. Poor clinical outcomes were characterized by modified Rankin Scale scores ranging from 3 to 6 at the 90‐day follow‐up. Multivariate logistic regression analysis was employed to examine the association between HHR values and posttreatment functional outcomes.

**Results:**

The study enrolled 353 participants (average age of 70.5 years with a standard deviation of 12.2 years; 218 were male), of whom 181 (51.3%) showed adverse clinical results after 90 days. Multivariate regression analysis revealed that higher HHR levels upon hospital admission independently predicted worse functional recovery (adjusted OR: 10.484; 95% confidence interval: 4.581–23.990; *p* = 0.001). Additional investigation using restricted cubic spline methodology confirmed a nonlinear, dose–dependent relationship between HHR values and negative prognosis likelihood (nonlinearity *p* value = 0.001). When implemented in a predictive framework, continuous HHR measurements exhibited a strong discriminatory capability, achieving a receiver operating characteristic curve value of 0.760 (95% CI: 0.710–0.810).

**Conclusion:**

Increased HHR levels demonstrate an independent correlation with poorer 90‐day recovery rates among ischemic stroke patients undergoing endovascular thrombectomy. This evidence positions HHR as a potentially valuable indicator for clinical prognosis assessment in such cases.

## 1. Introduction

Cerebral infarction caused by insufficient blood supply ranks among the primary causes of death and persistent impairment globally, creating substantial economic and social challenges for affected individuals, their relatives, and medical institutions across nations [1]. Mechanical clot removal (EVT) has become a fundamental treatment approach for sudden‐onset ischemic strokes resulting from blockages in major anterior circulation vessels, with extensive clinical data confirming its effectiveness in reestablishing cerebral perfusion and enhancing neurological recovery [2]. Although EVT techniques continue to advance, the incidence of poor outcomes following EVT remains at elevated levels [3].

Cerebrovascular ischemia and endovascular procedures are known to trigger systemic stress reactions that raise blood glucose levels. Research indicates that post‐EVT hyperglycemia may stimulate excessive superoxide generation, exacerbating blood–brain barrier (BBB) damage and reperfusion injuries, ultimately contributing to unfavorable clinical outcomes [4, 5]. Glycated hemoglobin (HbA1c), a well‐validated clinical indicator of chronic glucose regulation, plays a crucial role in diabetes management and risk evaluation [6]. Studies have demonstrated that elevated HbA1c values independently correlate with poorer prognosis after endovascular treatment [7]. Nevertheless, multiple biological variables can complicate HbA1c interpretation. Conditions like iron‐deficiency anemia may artificially increase HbA1c measurements through extended red blood cell lifespan, allowing more time for hemoglobin (HGB) glycation [8, 9]. Additionally, low preoperative HGB levels reflect compromised health status and correlate with higher complication rates and mortality risks. Postoperative complications following noncardiac surgical procedures have been linked to anemia [10]. Additionally, this condition may influence blood sugar regulation, particularly in cases where HbA1c measurements approach diagnostic cutoff values [11]. Inadequate glucose management can trigger oxidative damage, which, when coupled with reduced oxygen transport due to low HGB levels, creates a disparity between cellular oxygen requirements and availability. This dual mechanism potentially exacerbates clinical results for endovascular therapy recipients. The compounding negative impact implies that composite measures like the HbA1c‐to‐hemoglobin ratio (HHR) might offer a superior prognostic classification compared with individual biomarkers. Research indicates the HHR demonstrates enhanced predictive capacity for surgical outcomes in cardiac interventions [12]. Further investigations revealed elevated HHR levels correlated strongly with heightened risks of overall mortality and cardiac‐related deaths [13].

In cases of acute ischemic stroke, stress‐induced hyperglycemia frequently occurs during the acute phase. The stress hyperglycemia ratio (SHR), which compares acute blood glucose levels with long‐term glycemic control as measured by HbA1c, has proven to be a more reliable outcome indicator than isolated glucose or HbA1c measurements in certain situations [14, 15]. Similarly, the HHR was developed to enhance risk assessment by combining chronic glycemic data (HbA1c) with hematological factors (HGB levels).

Due to limited existing studies investigating HHR correlated with post‐EVT outcomes, this research implemented a retrospective cohort analysis to evaluate how HHR relates to 90‐day clinical results in acute ischemic stroke patients undergoing endovascular thrombectomy.

## 2. Subjects and Methods

### 2.1. Study Design and Population

**Figure 1:**
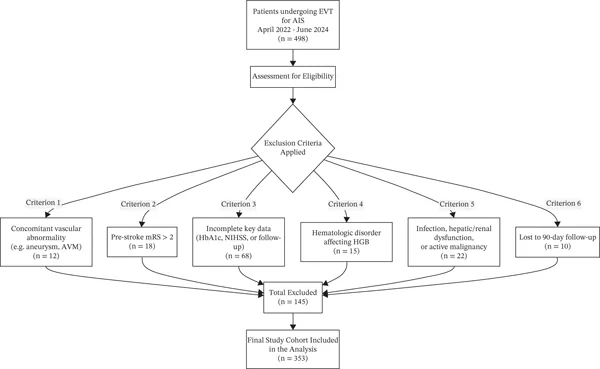
Flowchart of participant selection.

This research employed a single‐center retrospective cohort approach. Participants were selected from our Stroke Thrombectomy Registry, a prospectively maintained database that systematically recorded all cases of acute ischemic stroke caused by proximal anterior circulation occlusion treated with endovascular thrombectomy at Nanjing First Hospital from April 2022 to June 2024. The inclusion criteria consisted of: (1) Adult participants aged 18 years or older. (2) Confirmed diagnosis of anterior circulation ischemic stroke. (3) Treatment involving mechanical thrombectomy. (4) Documentation of both HbA1c levels (%) and HGB concentrations (g/dL) measured within the first day of hospitalization. (5) Availability of modified Rankin Scale (mRS) scores recorded during the 90‐day follow‐up period (allowing for a 14‐day variation before or after the target date). Exclusion criteria comprised: (1) Individuals presenting with concurrent diagnosed with specific cerebrovascular conditions including cerebral aneurysms, arteriovenous malformations, and moyamoya syndrome. (2) Those exhibiting functional impairment prior to stroke onset, as indicated by mRS scores exceeding 2 points. (3) Cases lacking comprehensive clinical records, diagnostic test results, or essential neuroimaging data for evaluation. (4) Subjects with documented blood disorders that substantially influence HGB concentrations, such as polycythemia vera, sickle cell anemia, acute hemorrhagic episodes necessitating blood replacement therapy before HGB assessment, or confirmed bone marrow dysfunction syndromes. (5) Participants presenting with infectious processes, severe hepatic or renal impairment (quantified by glomerular filtration rates below 30 mL/min/1.73m^2^ or serum creatinine levels surpassing 2.0 mg/dL), or currently undergoing treatment for malignant neoplasms. (6) Patients who discontinued participation prior to completing the 90‐day follow‐up evaluation. Figure 1 illustrates the participant selection process through a detailed flowchart.

### 2.2. Information Gathering

A team of qualified researchers methodically collected the study data through a uniform case documentation protocol, sourcing information from digital patient files and the specialized stroke database. The compiled personal details encompassed participants′ age and gender. Medical parameters incorporated recognized cardiovascular risk elements (elevated blood pressure, glucose metabolism disorders, dyslipidemia, ischemic heart conditions, irregular heart rhythms, cardiac insufficiency, tobacco use history, and prior cerebrovascular incidents), functional capacity before stroke occurrence (mRS evaluation), neurological deficit severity at hospital admission (NIHSS assessment), radiological observations (ASPECTS measurements), and underlying stroke causes according to the TOAST diagnostic. The diagnosis of high blood pressure followed these established benchmarks: three consecutive readings showing systolic/diastolic pressures exceeding 140/90 mmHg or ongoing prescription of blood pressure‐lowering drugs regardless of documented pressure values. Elevated lipid levels were characterized as total cholesterol surpassing 240 mg/dL or current lipid‐modifying therapy. The diagnostic criteria for hyperlipidemia included elevated total cholesterol (≥ 5.7 mmol/L), increased triglycerides (≥ 1.7 mmol/L), or raised low‐density lipoprotein cholesterol (≥ 3.6 mmol/L), along with patients receiving lipid‐modifying treatment. Diabetes diagnosis was established through fasting plasma glucose measurements (≥ 7.0 mmol/L) or active administration of antidiabetic drugs. Coronary artery disease was confirmed through medical records, diagnostic coronary imaging procedures (including angiographic or CT‐based assessments), or exercise testing outcomes. Hypertension was characterized by repeated clinical blood pressure readings exceeding 140/90 mmHg on three distinct occasions or the prescription of blood pressure‐lowering drugs. The definition of diabetes encompassed either fasting glucose levels at or above 7.0 mmol/L or the use of hypoglycemic medications. Lipid metabolism disorders were identified when patients exhibited cholesterol levels ≥ 5.7 mmol/L, triglyceride concentrations ≥ 1.7 mmol/L, LDL‐cholesterol values ≥ 3.6 mmol/L, or were undergoing pharmacological management for lipid abnormalities. The treatment protocol encompassed the administration of intravenous thrombolytic therapy, along with the evaluation of collateral blood flow and the precise measurement of the interval between symptom manifestation and vessel reopening. Collateral circulation was classified on a 0–4 scale following the ASITN/SIR guidelines, where Scores 0–1 indicated inadequate collateral flow, whereas Scores 2–4 represented satisfactory to optimal circulation [16]. Effective restoration of blood flow was characterized by achieving mTICI scores between 2b and 3 [17]. Two medical practitioners, unaware of patient outcomes, separately reviewed all angiographic and CT scan results. In cases of disagreement, a third expert was engaged to reach consensus. Blood specimens were collected within 1 day of hospital admission and processed using standardized laboratory equipment to measure various hematological indices, such as white blood cell count, neutrophil levels, and HGB concentration, in addition to other essential blood parameters. Blood sugar concentrations were quantified, with HbA1c levels determined through high‐performance liquid chromatography techniques. The HHR was calculated by dividing the percentage of HbA1c by HGB concentration in grams per deciliter. Ethical clearance for this investigation was obtained from the Nanjing First Hospital′s Institutional Review Board and Ethics Committee. Due to the retrospective nature of the study and the utilization of completely de‐identified patient information gathered through routine clinical practice, the requirement for participant consent was exempted. All research activities strictly complied with the ethical principles outlined in the Helsinki Declaration.

#### 2.2.1. Outcome Assessment

Functional outcomes were systematically evaluated at the 90‐day mark by board‐certified neurologists who remained blinded to laboratory findings. These assessments were performed through structured telephone conversations or face‐to‐face consultations. The main endpoint of interest—unfavorable functional status—was characterized by mRS scores ranging from 3 to 6, indicating moderate‐to‐severe impairment or mortality.

### 2.3. Statistical Analysis

Continuous variables following a normal distribution are presented using means accompanied by standard deviations, whereas those not conforming to normal distribution are displayed as medians with interquartile ranges. Categorical variables were presented as counts with corresponding percentages. Study participants were categorized based on their 90‐day functional status, dividing them into two distinct groups: those with favorable outcomes (mRS Scores 0–2) and those with unfavorable outcomes (mRS Scores 3–6). Comparative analyses between these groups utilized the Student′s *t*‐test or Mann–Whitney *U* test for continuous variables, whereas categorical data were examined using either the chi‐square test or Fisher′s exact test, depending on the data characteristics. The potential association between HHR and poor functional outcomes was investigated through binary logistic regression analysis. Multivariate models incorporated adjustments for age, sex, and all variables that demonstrated a univariate association (*p* < 0.1), including time from symptom onset to recanalization, baseline NIHSS score, collateral circulation status, reperfusion success, leukocyte levels, neutrophil counts, and blood glucose levels. Additionally, a restricted cubic spline model (with knots positioned at the 5th, 50th, and 95th percentiles) was employed to explore potential nonlinear relationships between HHR and functional outcomes, while maintaining adjustments for the same set of covariates. The model′s predictive accuracy and discriminative ability were evaluated using The Hosmer–Lemeshow goodness‐of‐fit test and receiver operating characteristic (ROC) curve analysis were employed to evaluate model performance. Statistical significance was determined using a two‐tailed *p* value threshold of 0.05. All statistical computations were conducted using SPSS Version 27.0 and R software Version 4.5.0.

## 3. Results

### 3.1. Initial Demographic and Clinical Features

Following the evaluation of 498 potential participants, 353 individuals met the inclusion criteria and were included in the final study population (Figure 1). The study group exhibited an average age of 70.5 years, with male participants constituting 61.8% of the sample. Initial clinical assessments revealed median values of 13.0 (interquartile range 10.0–16.0) for NIHSS scores and 9.0 (interquartile range 8.0–10.0) for ASPECTS measurements. Intravenous thrombolytic therapy had been administered to 36.0% of cases, with successful vascular recanalization occurring in 94.4% of treated patients. Comprehensive baseline data encompassing demographic, clinical, and imaging parameters are documented in Table 1. Pre‐thrombectomy evaluations identified an inadequate collateral blood flow in 56.1% of subjects. Statistical analysis demonstrated significant variations in age, white blood cell levels, and neutrophil counts when stratified by HHR quartiles. Additionally, the incidence of both diabetes mellitus and coronary artery disease exhibited a progressive increase corresponding to higher HHR quartile classifications (Table 2).

**Table 1:** Baseline characteristics stratified by the quartile of HHR score.

Various	HHR				
	First quartile, *N* = 88	Second quartile, *N* = 88	Third quartile, *N* = 88	Fourth quartile, *N* = 89	*p*
Age, years	63.9 ± 13.6	70.1 ± 12.8	73.4 ± 10.1	74.5 ± 9.1	0.002
Male, *n* (%)	56 (63.6)	57 (64.8)	53 (60.2)	52 (58.4)	0.807
Hypertension, *n* (%)	62 (69.7)	67 (77.0)	68 (76.4)	70 (79.5)	0.412
Diabetes mellitus, *n* (%)	6 (6.7)	21 (24.1)	39 (43.8)	60 (68.2)	0.001
Hyperlipidemia, *n* (%)	10 (11.4)	12 (13.6)	8 (9.1)	11 (12.4)	0.813
Coronary heart disease, *n* (%)	7 (7.9)	13 (14.9)	16 (18.0)	23 (26.1)	0.014
Atrial fibrillation, *n* (%)	35 (39.8)	37 (42.0)	44 (50.0)	34 (38.2)	0.396
Heart failure, *n* (%)	6 (6.8)	7 (8.0)	11 (12.5)	9 (10.1)	0.581
Smoking, *n* (%)	28 (31.8)	32 (36.4)	30 (34.1)	33 (37.1)	0.882
Systolic blood pressure, mmHg	139.0 (127.5, 151.0)	139.0 (124.0, 147.0)	146.0 (130.0, 160.5)	141.0 (128.3, 157.8)	0.064
Diastolic blood pressure, mmHg	85.5 (76.3, 98.8)	85.0 (74.0, 94.0)	83.5 (74.3, 94.0)	86.0 (75.5, 93.0)	0.772
Time from onset to recanalization, min	326.0 (236.0, 483.3)	335.5 (235.8, 639.3)	349.0 (253.0, 527.3)	352.0 (272.5, 519.5)	0.592
Baseline NIHSS, score	12.0 (9.5, 15.0)	12.0 (9.0, 17.0)	14.0 (11.0, 17.0)	14.0 (9.0, 16.8)	0.698
Baseline ASPECTS, score	9 (8.0, 9.0)	9 (8.0, 10.0)	9 (8.0, 10.0)	9 (8.0, 10.0)	0.497
Cause of stroke, *n* (%)					0.154
Atherosclerotic	47 (53.4)	43 (48.9)	32 (36.4)	41 (46.2)	
Cardioembolic	29 (33.0)	39 (44.3)	47 (53.4)	40 (44.9)	
Others	12 (13.6)	6 (6.8)	9 (10.2)	8 (9.0)	
Prior IVT treatment, *n* (%)	32 (36.0)	35 (40.2)	30 (33.7)	36 (40.9)	0.708
Poor collateral circulation, *n* (%)	48 (53.9)	49 (56.3)	47 (52.8)	54 (61.4)	0.578
Successful reperfusion, *n* (%)	84 (94.4)	83 (95.4)	85 (95.5)	78 (88.6)	0.214
Vascular occlusion site, *n* (%)					0.740
Middle cerebral artery	55 (62.5)	58 (65.9)	62 (70.5)	59 (66.3)	
Internal carotid artery	33 (37.5)	30 (34.1)	26 (29.5)	30 (33.7)	
Laboratory data					
Leucocyte count, ×10^9^	10.1 ± 2.9	9.2 ± 3.0	9.1 ± 3.4	10.0 ± 3.9	0.051
Neutrophil count, ×10^9^	8.2 ± 2.6	7.4 ± 2.8	7.5 ± 3.4	8.2 ± 3.7	0.042
Glycemia, mmol/L	6.3 ± 1.5	6.5 ± 1.9	7.3 ± 2.2	8.5 ± 2.9	0.001
HGB, g/dL	14.3 ± 1.2	12.9 ± 1.3	12.0 ± 1.3	10.9 ± 1.9	0.001
HbA1c, *n* (%)	5.7 (5.4, 5.9)	6.0 (5.7, 6.3)	6.4 (5.9, 6.9)	7.3 (6.5, 8.1)	0.001
HHR	0.39 ± 0.03	0.47 ± 0.02	0.53 ± 0.03	0.69 ± 0.11	0.001

Abbreviations: ASPECTS, the Alberta Stroke Program Early Computed Tomography Score; HGB, hemoglobin; HHR, HbA1c‐hemoglobin ratio; IVT, intravenous thrombolysis; NIHSS, National Institute of Health Stroke Scale.

**Table 2:** Clinical Characteristics of Study Participants According to Patients with Poor

Various	Study sample (*n* = 353)	Poor outcome at 90 days		*p*
		Yes (*n* = 181), no (*n* = 172)		
Age, years	70.5 ± 12.2	72.3 ± 11.1	68.2 ± 12.9	0.001
Male, *n* (%)	218 (61.8)	104 (60.5)	114 (63.0)	0.627
Hypertension, *n* (%)	267 (75.6)	141 (77.9)	126 (73.3)	0.310
Diabetes mellitus, *n* (%)	126 (35.7)	71 (39.2)	56 (32.0)	0.155
Hyperlipidemia, *n* (%)	41 (11.6)	21 (11.6)	20 (11.6)	0.994
Coronary heart disease, *n* (%)	59 (16.7)	34 (18.8)	25 (14.5)	0.285
Atrial fibrillation, *n* (%)	150 (42.5)	83 (45.9)	67 (39.0)	0.493
Heart failure, *n* (%)	33 (9.3)	19 (10.5)	14 (8.1)	0.447
Smoking, *n* (%)	123 (34.8)	70 (56.9)	53 (43.1)	0.121
Systolic blood pressure, mmHg	141.0 (127.5, 153.0)	140.0 (127.5, 155.0)	141.5 (127.3, 152.0)	0.680
Diastolic blood pressure, mmHg	85.0 (76.0, 94.0)	84.0 (74.0, 94.0)	86.0 (77.0, 94.0)	0.492
Time from onset to recanalization, min	344.0 (246, 514.0)	354.0 (265.5, 554.0)	322.5 (223.0, 466.5)	0.001
Baseline NIHSS, score	13.0 (10.0, 16.0)	14.0 (11.0, 18.0)	12.0 (8.0, 15.0)	0.001
Baseline ASPECTS, score	9.0 (8.0, 10.0)	9.0 (8.0, 10.0)	9.0 (8.0, 10.0)	0.336
Cause of stroke, *n* (%)				0.227
Atherosclerotic	163 (46.2)	79 (43.6)	84 (48.8)	
Cardioembolic	155 (43.9)	87 (48.1)	68 (39.5)	
Others	35 (9.9)	15 (8.3)	20 (11.6)	
Prior IVT treatment, *n* (%)	122 (34.6)	66 (36.5)	67 (39.0)	0.629
Poor collateral circulation, *n* (%)	198 (56.1)	110 (60.8)	88 (51.2)	0.069
Successful reperfusion, *n* (%)	330 (93.5)	162 (90.1)	167 (97.1)	0.007
Vascular occlusion site, *n* (%)				0.497
Middle cerebral artery	234 (66.3)	123 (68.0)	111 (64.5)	
Internal carotid artery	119 (33.7)	58 (32.0)	61 (35.5)	
Laboratory data				
Leucocyte count, ×10^9^	9.6 ± 3.3	10.0 ± 3.7	9.1 ± 2.8	0.012
Neutrophil count, ×10^9^	7.8 ± 3.1	8.3 ± 3.5	7.3 ± 2.6	0.004
Glycemia, mmol/L	7.2 ± 2.4	7.6 ± 2.6	6.5 ± 1.9	0.001
HGB, g/dL	12.3 ± 1.9	12.1 ± 1.9	13.0 ± 1.8	0.001
HbA1c, *n* (%)	6.2 (5.7, 6.9)	6.5 (5.9, 7.2)	5.9 (5.6, 6.4)	0.001
HHR	0.52 ± 0.12	0.57 ± 0.14	0.47 ± 0.08	0.001

Abbreviations: ASPECTS, the Alberta Stroke Program Early Computed Tomography Score; HGB, hemoglobin; HHR, HbA1c‐hemoglobin ratio; IVT, intravenous thrombolysis; NIHSS, National Institute of Health Stroke Scale.

### 3.2. Relationships of HHR With Treatment Results

**Figure 2:**
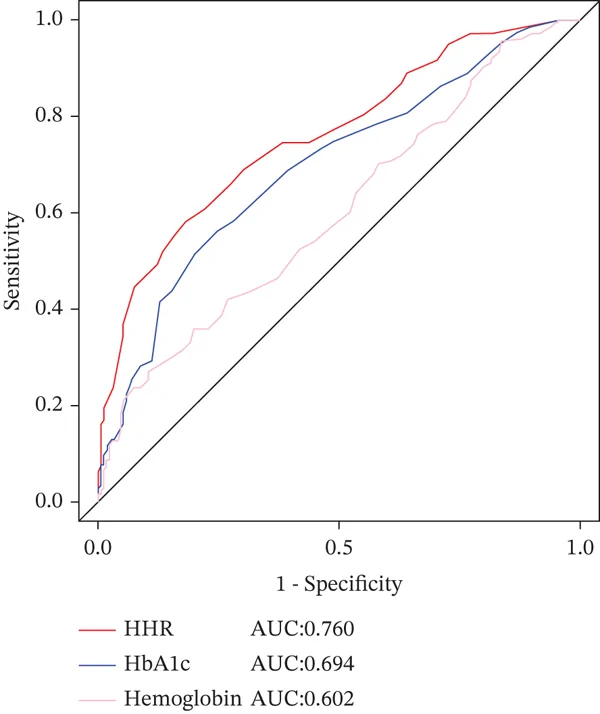
ROC curves for HHR and Hba1c.

**Figure 3:**
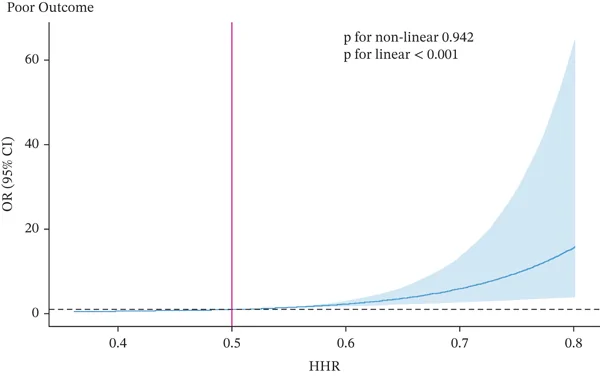
Association was fitted with restricted cubic spline with 3 knots (at 5th, 55th, 95th percentiles) adjusted for covariates included in model 3 in Table 3.

Over the 3‐month follow‐up duration, 181 participants (51.3%) demonstrated suboptimal functional recovery. Table 1 displays the comparative analysis of demographic, biochemical, and radiological features between patients with adverse and positive outcomes. Initial statistical evaluation revealed that individuals experiencing unfavorable outcomes were notably more advanced in age compared with their counterparts with better recovery (average age 72.3 vs. 68.2 years; *p* = 0.001). Additionally, the cohort with poor outcomes displayed elevated median NIHSS scores at baseline (14.0 compared with 10.0; *p* = 0.002), extended median duration from symptom onset to vessel reopening (354.0 vs. 322.5 min; *p* = 0.001), and reduced successful blood flow restoration rates (90.1% vs. 97.1%; *p* = 0.007). Laboratory examinations showed that patients with worse outcomes had increased white blood cell levels (mean 10.0 vs. 9.1; *p* = 0.012), higher neutrophil values (mean 8.3 vs. 7.3; *p* = 0.004), and elevated blood glucose concentrations (mean 7.6 vs. 6.5). Statistical analysis revealed a highly significant difference (*p* < 0.001) between groups with poor and favorable outcomes. Patients experiencing adverse clinical results showed substantially greater baseline HHR values (mean 0.57) compared with those with positive outcomes (mean 0.47). Multivariate regression analysis established HHR as an independent predictor of worse 90‐day prognosis after endovascular treatment (adjusted OR 10.484; 95% CI 4.581–23.990). The predictive model displayed appropriate goodness‐of‐fit (Hosmer–Lemeshow *p* = 0.744), with variance inflation factors all below the threshold of 5, confirming the absence of problematic multicollinearity. ROC analysis indicated superior predictive performance for HHR (AUC 0.760, 95% CI 0.711–0.809) relative to both HAb1c (AUC 0.697, 95% CI 0.642–0.751) and HGB‐based models (AUC 0.602). The 95% confidence interval (0.534–0.661) demonstrated a statistically significant enhancement in prediction accuracy (Figure 2). Restricted cubic spline analysis revealed a clear linear correlation between HHR values and unfavorable neurological outcomes at 90 days (*p* value for linear trend = 0.001; Figure 3). Stratified analyses according to diabetes status were performed, and the results are presented in Table S1.

**Table 3:** Binary regression analysis for the association between HHR score and poor outcome at 3 months.

Variables	Model 1 OR (95% CI)	*p*	Model 2 OR (95% CI)	*p*	Model 3 OR (95% CI)	*p*
HHR (per one‐unit increase)	8.291 (6.076, 11.054)	0.001	8.032 (5.843, 10.948)	0.001	8.330 (5.911, 12.614)	0.001
HHR quartile						
First	Reference		Reference		Reference	
Second	1.651(0.884–3.083)	0.116	1.014 (0.994–1.035)	0.199	1.684 (0.837–3.391	0.114
Third	2.734(1.470–5.087)	0.001	2.441 (1.280–4.654)	0.021	2.502 (1.226–5.105)	0.012
Fourth	10.100 (5.019–20.324)	0.001	8.965 (4.342, 18.509)	0.001	10.484 (4.581–23.990)	0.001

*Note:* Model 1: Multivariate regression analysis adjusted for age and gender. Model 2: Multivariate regression analysis adjusted for age, gender, time from onset to recanalization, baseline NIHSS score, and poor collateral status. Model 3: Multivariate regression analysis adjusted for age, gender, and variables with a *p* value < 0.1 in the univariate analysis including time from onset to recanalization, baseline NIHSS score, poor collateral status, successful reperfusion, leucocyte count, neutrophil count, and glycemia and HHR levels.

Abbreviations: CI, confidence interval; HHR, HbA1c‐hemoglobin ratio; OR, odds ratio.

## 4. Discussion

This investigation established high HHR levels as a robust prognostic indicator for adverse clinical outcomes post‐EVT, independent of multiple covariates including demographic factors (age and sex), treatment parameters (recanalization time and reperfusion success), and baseline characteristics (NIHSS score, collateral circulation status, inflammatory markers, and blood glucose levels).

Both cerebrovascular accidents and endovascular therapy may activate the body′s stress response, leading to heightened secretion of counterregulatory hormones including cortisol and catecholamines. These hormones elevate blood sugar levels by opposing insulin′s effects [18]. During severe illness, the combination of insulin resistance and impaired pancreatic function can further reduce insulin production [19]. Research has demonstrated connections between insulin resistance, *β*‐cell dysfunction, and both large artery and small vessel cerebrovascular events [20]. HbA1c serves as a standard clinical indicator of recent glucose control. Elevated HbA1c levels reflect pancreatic dysfunction in individuals diagnosed with Type 2 diabetes based on this marker [21]. A cohort investigation revealed that higher HbA1c values independently predict poorer outcomes after mechanical thrombectomy procedures [7]. Additionally, HGB, as the oxygen‐carrying protein in red blood cells, plays a crucial role in maintaining the balance between oxygen delivery and tissue demand. Disruption of this equilibrium forms the fundamental pathological basis of acute cerebral ischemia. Studies have shown that anemia correlates with more severe stroke [22] and plays a crucial role in patient outcomes. Research demonstrates that patients presenting with anemia during hospitalization face greater mortality risks within the initial 3‐year poststroke period when compared with individuals with standard HGB concentrations [23]. Since persistent high blood sugar levels (measured through HbA1c) and low HGB independently worsen prognosis via shared mechanisms including oxidative damage and vascular impairment, their interaction might produce amplified negative effects. Considering HGB′s impact on HbA1c measurements [8], the HHR potentially enhances prognostic accuracy for endovascular therapy outcomes by simultaneously evaluating these dual risk factors. Previous investigations have established HHR′s predictive value for adverse events in diabetic patients undergoing noncardiac surgical procedures [24]. This research builds upon existing knowledge about HHR′s detrimental effects in nonsurgical contexts by revealing its strong correlation with unfavorable results after endovascular interventions. Additionally, the strategy of enhancing traditional biological markers through Research has demonstrated the clinical significance of the SHR, a metric that evaluates acute glucose levels while considering long‐term glycemic control as reflected by HbA1c. This parameter has proven particularly valuable in predicting stroke prognosis, outperforming conventional measures [14, 15]. Similarly, the HHR enhances the interpretation of HbA1c values by incorporating hemoglobin concentrations, thereby mitigating potential distortions caused by hematological variations.

The precise pathophysiological processes by which HHR affects outcomes after EVT in ischemic stroke remain incompletely understood, though several potential mechanisms have been proposed. One primary contributor involves the elevated oxidative stress characteristic of diabetic conditions, which causes widespread harm to essential cellular components including proteins, lipid membranes, and genetic material [25, 26]. The impact of anemia on cerebral circulation extends beyond mere oxygen deprivation, involving multiple factors. This condition may induce oxidative stress when the disparity between oxygen supply and metabolic requirements worsens neural injury, cognitive impairment, and susceptibility to cerebrovascular events [27]. Such pathological changes could increase the risk of oxidative damage even after successful vessel reopening through EVT [4, 28]. Another significant factor relates to the connection between elevated HbA1c levels and impaired endothelial function [28]. Persistent hyperglycemia may stimulate reactive oxygen species (ROS) production while diminishing nitric oxide availability, thereby compromising vascular endothelial integrity [29]. In individuals with diabetes, these alterations could compromise endothelial function and are often observed alongside increased vascular fragility and BBB deterioration, which elevate the risk of developing cerebral edema and experiencing reperfusion‐related damage [30, 31]. Persistent anemia contributes to worsening endothelial dysfunction via mechanisms involving chronic inflammatory responses, enhanced inducible nitric oxide synthase (iNOS) activity, and excessive ROS generation in vascular tissues [32]. These pathological changes result in fibrotic thickening of arterial walls, which subsequently diminishes vasodilatory capacity and predisposes to small vessel occlusion and thrombus formation [33]. Within the cerebral vasculature, this diminished vasodilation capacity interferes with normal autoregulatory processes, ultimately decreasing cerebral perfusion [34]. Furthermore, these vascular abnormalities contribute to BBB integrity loss [33]. Moreover, elevated HbA1c levels serve as a clinical marker for persistent hyperglycemia, reflecting long‐term metabolic disturbances that contribute to cerebrovascular injury. The pathophysiological processes involved are primarily associated with chronic exposure to high glucose concentrations and may operate independently from the thromboinflammatory mechanisms triggered during episodes of acute stress‐induced hyperglycemia [35, 36]. Disruptions in metabolic processes can trigger inflammatory responses. Our research aligns with existing evidence showing that inflammatory indicators, such as the neutrophil‐to‐albumin ratio (NPAR)—a composite measure of immune cell activity and nutritional condition—serve as reliable predictors of functional recovery following endovascular therapy. These observations reinforce the concept of an interconnected pathological mechanism combining metabolic dysfunction with widespread inflammation [37]. Anemia resulting from iron deficiency enhances thrombotic risk, mainly by inducing excessive platelet production and creating a state of heightened coagulation [38]. Such thrombotic predisposition arises from altered blood flow characteristics due to decreased red blood cell flexibility and increased blood thickness in microcytic anemia [39]. Furthermore, the co‐occurrence of elevated HbA1c levels and reduced HGB frequently indicates underlying systemic health issues that may worsen stroke outcomes [40].

This investigation, while establishing a correlation between HHR and unfavorable outcomes after EVT, presents certain methodological constraints. Primarily, the retrospective nature of the research design introduces possible selection bias. The study population was restricted to Chinese individuals experiencing anterior circulation strokes, potentially restricting the applicability of findings to other demographic groups or stroke categories. Given variations in cultural practices, dietary patterns, and healthcare infrastructures across different nations and regions, the broader relevance of our conclusions may be constrained. Subsequent research involving multiple centers and diverse ethnic and geographical cohorts would be necessary to confirm the universal applicability of HHR findings. Furthermore, the collection of blood specimens immediately following stroke onset implies that HHR measurements might reflect acute physiological stress reactions rather than representing typical baseline values. This temporal factor could compromise the accuracy of these biomarkers as indicators of prestroke conditions. Additionally, prior research has identified a connection between inactive behavior combined with unhealthy eating habits and increased susceptibility to Type 2 diabetes [41]. This highlights the need for comprehensive lifestyle assessments in future studies to better understand the multifaceted influences on post‐EVT outcomes. Although rigorous selection criteria were applied, the potential for remaining confounding variables cannot be completely eliminated. This includes factors such as glycemic control methods, the degree of glucose regulation achieved, and underlying reasons for anemia. Additional considerations involve prestroke diabetes treatment regimens, the exact nature of anemia causes, and meticulous glycemic and comorbidity management throughout the 3‐month observation period. These unaccounted elements might impact both HHR measurements and extended prognosis. Furthermore, although our multicollinearity analysis showed no concerning patterns, the notably elevated odds ratio warrants careful consideration and independent verification to exclude potential model overfitting.

## 5. Conclusions

The research demonstrates that increased HHR levels serve as a reliable indicator for predicting adverse clinical results within 90 days among individuals undergoing endovascular treatment for ischemic stroke. These results highlight the necessity for comprehensive, multi‐institutional longitudinal studies to validate its effectiveness in clinical prognosis assessment and investigate the underlying biological mechanisms responsible for this association.

## Funding

No funding was received for this manuscript.

## Conflicts of Interest

The authors declare no conflicts of interest.

## Supporting information


**Supporting Information** Additional supporting information can be found online in the Supporting Information section. Stratified analysis based on diabetes status (Table S1) confirmed HHR′s consistent predictive value across both subgroups, with particularly pronounced effects observed in diabetic patients.

## Data Availability

The original datasets used in this research can be obtained by contacting the corresponding authors upon reasonable request, with no unnecessary restrictions.
