# Supplementary material for: Higher HbA1c‐Hemoglobin Ratio Was Associated With Poor Outcome in Endovascular Thrombectomy Patients
Source: Behav Neurol. 2026 Mar 13;2026:6668235. doi: 10.1155/bn/6668235 (PMC13580790; doi:10.1155/bn/6668235)
Supplement: Supplementary file 1 [file BN-2026-6668235-s001.docx]

Supplementary Table 1

HHR (poor outcome vs. good outcome)

|  | n | OR | 95%CI | P for interaction=0.066 |
| --- | --- | --- | --- | --- |
| With diabetes | 127 | 12.8 | 8.38-24.89 |  |
| Without diabetes | 226 | 7.80 | 3.81-14.09 |  |

Multivariate regression analysis adjusted for age, gender and variables with a P value < 0.1 in the univariate analysis including Time from onset to recanalization, baseline NIHSS score, poor collateral status, successful reperfusion, leucocyte count, neutrophil count, glycemia and HHR levels.
